# Scaffold-Free Tubular Engineered Heart Tissue From Human Induced Pluripotent Stem Cells Using Bio-3D Printing Technology *in vivo*

**DOI:** 10.3389/fcvm.2021.806215

**Published:** 2022-01-20

**Authors:** Yujiro Kawai, Shugo Tohyama, Kenichi Arai, Tadashi Tamura, Yusuke Soma, Keiichi Fukuda, Hideyuki Shimizu, Koichi Nakayama, Eiji Kobayashi

**Affiliations:** ^1^Department of Cardiovascular Surgery, Keio University School of Medicine, Tokyo, Japan; ^2^Department of Cardiology, Keio University School of Medicine, Tokyo, Japan; ^3^Department of Regenerative Medicine and Biomedical Engineering, Saga University, Saga, Japan; ^4^Department of Organ Fabrication, Keio University School of Medicine, Tokyo, Japan; ^5^Department of Kidney Regenerative Medicine, The Jikei University School of Medicine, Tokyo, Japan

**Keywords:** human-induced pluripotent stem cell, engineered heart tissue, bio-3D bioprinting, tubular tissue, cardiomyocyte

## Abstract

Engineered heart tissues (EHTs) that are fabricated using human induced pluripotent stem cells (hiPSCs) have been considered as potential cardiac tissue substitutes in case of heart failure. In the present study, we have created hiPSC-derived cardiac organoids (hiPSC-COs) comprised of hiPSC-derived cardiomyocytes, human umbilical vein endothelial cells, and human fibroblasts. To produce a beating conduit for patients suffering from congenital heart diseases, we constructed scaffold-free tubular EHTs (T-EHTs) using hiPSC-COs and bio-3D printing with needle arrays. The bio-3D printed T-EHTs were cut open and transplanted around the abdominal aorta as well as the inferior vena cava (IVC) of NOG mice. The transplanted T-EHTs were covered with the omentum, and the abdomen was closed after completion of the procedure. Additionally, to compare the functionality of hiPSC-COs with that of T-EHTs, we transplanted the former around the aorta and IVC as well as injecting them into the subcutaneous tissue on the back of the mice. After 1 m of the transplantation procedures, we observed the beating of the T-EHTs in the mice. In histological analysis, the T-EHTs showed clear striation of the myocardium and vascularization compared to hiPSC-COs transplanted around the aorta or in subcutaneous tissue. Based on these results, bio-3D-printed T-EHTs exhibited a better maturation *in vivo* as compared to the hiPSC-COs. Therefore, these beating T-EHTs may form conduits for congenital heart disease patients, and T-EHT transplantation can form a treatment option in such cases.

## Introduction

Heart failure is a fatal condition, and its treatment is limited to heart transplantation. However, in recent times, engineered heart tissues (EHTs) or cardiac organoids (COs) that are fabricated using human induced pluripotent stem cells (hiPSCs) have been considered as potential cardiac tissue substitutes in heart failure patients. These EHTs may be developed in different ways. Incidentally, the EHTs with a thickness contract are stronger than COs. Additionally, histological analysis shows that EHTs are mature tissues, but in comparison to adult human heart tissue, they are considered immature. Since these EHTs respond to drugs like a normal human heart tissue or diseased heart tissue, they may form a promising candidate for drug screening in new drug development programs (1). In fact, in certain preclinical studies using animal models, hiPSC-derived EHTs or hiPSC-derived COs (hiPSC-COs) have been developed and transplanted (2, 3), wherein the transplanted hiPSC-COs have successfully recovered injured heart function (2). Neovascularization of the EHTs originate from the host heart, and this is important for their long-term survival *in vivo*. In fact, for better neovascularization of the EHTs, endothelial cells can be included in their development process, thereby resulting in better engraftment.

Tubular tissues can be created using a bio-3D printer. Artificial vascular grafts have already been used clinically for aortic surgery, as a bypass graft in peripheral artery disease, as a conduit in congenital heart surgery, and for vascular access in hemodialysis. However, artificial vascular grafts, especially the ones with small diameters, have many problems, such as being prone to infection and thrombosis. A recent study has reported the fabrication of tubular tissues with the help of a bio-3D printer and without the use of any biomaterials (4). These bio-3D-printed tubular tissues can help to overcome the problems associated with artificial grafts. Therefore, the tubular EHTs (T-EHTs) can form a substitute for synthetic vascular grafts. For instance, a beating T-EHT could function as a conduit during congenital heart surgery, provide vascular access, or act as a left ventricular assist device in case of low left ventricle ejection fraction, similar to an intra-aortic balloon pump where the tubular heart tissue beats in the descending aorta.

Previously, we have reported the development of an efficient, large-scale culture system for obtaining hiPSC-derived cardiomyocytes (hiPSC-CMs) with high purity (5–7). We co-cultured these purified hiPSC-CMs with human umbilical vein endothelial cells (HUVECs) and normal human dermal fibroblasts (NHDFs) in 96-well U-bottom plates to produce hiPSC-COs. Thereafter, using these hiPSC-COs and a bio-3D printer with needle arrays, we created scaffold-free T-EHTs. Finally, our bio-3D-printed T-EHTs were ready without the use of any biomaterials, such as Matrigel or fibrin gel as the scaffolds (8). In this study, we fabricated scaffold-free T-EHTs with hiPSC-COs using bio-3D printing with needle arrays, transplanted these T-EHTs into NOG mice, and assessed their beating and histological characteristics.

## Materials and Methods

### Production of hiPSC-CMs

To produce cardiomyocytes, we used hiPSCs (253G4) that were maintained on growth factor-reduced Matrigel-coated culture plates using modified StemFit media (Ajinomoto, Tokyo, Japan) (6, 9). The cardiomyocytes differentiated from the hiPSCs, as reported in a previous study (6). Subsequently, the hiPSC-CMs were purified by metabolic selection using StemFit medium AS501 (Ajinomoto, Tokyo, Japan) (5, 7) and cryopreserved (6).

### Production of hiPSC-COs

First, we created spherical hiPSC-COs. For this, we prepared cryopreserved, purified hiPSC-CMs, HUVECs (Lonza Inc., Walkersville, MD, USA), and NHDFs (Lonza Inc.). These cells were cultured in 5% fetal bovine serum/minimum essential medium-α (MEM-α, Thermo Fisher Scientific, Inc., Waltham, MA, USA), endothelial basal medium-2 (Lonza Inc.), and fibroblast growth basal medium-2 (Lonza Inc.), respectively. Thereafter, these cells were seeded at various ratios, such as 100:0:0, 80:10:10, 60:20:20, and 50:25:25 of hiPSC-CMs:HUVECs:NHDFs, within ultra-low attachment 96 U-well plates (SUMITOMO BAKELITE, Tokyo, Japan) to form spherical COs. Each CO included 3.0 × 10^4^ cells. The beating rate and contraction of the four types of organoids generated from the four different cell mixtures were tested 10 d after culture. These cardiac spheroids were stained with 12.5 μM XenoLight DiR membrane dye (Far-red, Caliper Life Sciences, MA, USA), according to the manufacturer's instructions.

### Creation of T-EHTs Using Bio-3D Printing Technology

Among the four types of cell suspensions, the one composed of 50% hiPSC-CMs, 25% HUVECs, and 25% NHDFs was seeded within ultra-low attachment 96 U-well plates (SUMITOMO BAKELITE) to form hiPSC-COs, such that there were 30,000 cells per well. This cell suspension was cultured for 5 d to promote spherical CO formation. Previously, we had reported a cardiac construct fabrication method using a bio-3D printer (Cyfuse Biomedical K.K., Tokyo, Japan) (10). The hiPSC-COs consisting of 50% hiPSC-CMs, 25% HUVECs, and 25% NHDFs were loaded on a 9 × 9 needle array, according to the 3D tubular design (10 layers), and cultured in a bioreactor for 7 d to promote fusion of the hiPSC-COs in the cardiac constructs. Finally, the T-EHTs fabricated in the needle array were removed and used for transplantation in animals.

### Transplantation

#### Experimental Animals

We used 7-w-old adult male NOG mice (NOD/Shi-scid-IL2 receptor gamma-null, CLEA Japan, Inc.) for *in vivo* testing of our T-EHTs. The experimental protocol was approved by the Institutional Animal Care and Use Committee, Keio University. All experimental studies were performed in accordance with the Institutional Guidelines on Animal Experimentation at Keio University and the Fundamental Guidelines for Proper Conduct of Animal Experiments and Related Activities in Academic Research Institutions (Ministry of Education, Culture, Sports, Science and Technology). All the animals received humane care consistent with the Guide for the Care and Use of Laboratory Animals.

#### Transplantation Procedure

The mice were anesthetized prior to the laparotomy procedure. The abdominal aorta (AA) and inferior vena cava (IVC) were exposed and separated from the retroperitoneal membrane. The bio-3D printed tubular tissue was cut open, and the AA and IVC were wrapped in it. Ultimately, the tubular tissue was covered with the omentum, and the abdomen was closed. Additionally, the hiPSC-COs were transplanted into the subcutaneous and retroperitoneal tissues of the NOG mice and covered with the omentum to compare their functionality with that of the T-EHTs.

### Analysis of Engraftment

We opened the abdomen of the NOG mice and observed the transplanted tissues using bioluminescent imaging (NightOWL LB983, Berthold Technologies, Germany), which can detect tissues labeled with far-red, after 1 w as well as 1 m of the transplantation procedure (11). The T-EHT in a mouse was explanted 1 w post-transplantation for histological testing based upon the far-red fluorescence. The hiPSC-COs and T-EHTs in mice were explanted in the same way 1 m post-transplantation, and the T-EHTs were assessed by beating analysis and histological analysis. We only analyzed the beating of the T-EHTs because of the difficulty in detecting beats in the explanted hiPSC-COs which were buried in surrounding tissues.

### Beating Analysis and Electrical Stimulation

To visualize the contraction and analyze the beating rate of the transplanted cardiac constructs, we recorded videos of these constructs using a digital camera (Leica MC120 HD, Leica Microsystems Inc., Buffalo Grove, IL, USA) mounted on a stereoscopic microscope SZX7 (Olympus, Tokyo, Japan). The recorded movies were analyzed using a laboratory-developed software program that could recognize the cardiac construct and track the extent of movement (1). In analysis, several points of the explanted T-EHT were tracked using a digital camera and their movements were recorded. The amounts of movement of the T-EHT were shown in pixels. Beating analysis and electrical stimulation were only performed on the T-EHT.

The mice were sacrificed 1 m post-transplantation. The cardiac constructs were removed from the AA, incubated in the medium for 30 min at 37°C, and finally transferred to a chamber with two platinum rods connected to an electrical stimulation device (8). Thereafter, these T-EHTs were stimulated with bipolar electrical pulses of 15 V and 1 Hz or 2 Hz for 10 ms (repeated every 990 ms or 490 ms, respectively), and the data were analyzed using a laboratory-developed software program.

### Histology

The T-EHT samples were fixed with 4% paraformaldehyde for 48 h at 4°C, embedded in paraffin, and cut into 4 μm thick sections. All the sections were subjected to hematoxylin and eosin (HE) and immunostaining. The primary antibodies used in the immunostaining procedure included myosin light chain 2a (MLC2a; dilution 1:100, Synaptic Systems GmbH, Goettingen, Germany), MLC2v (dilution 1:100, Abcam Plc., Cambridge, UK), cardiac troponin T (cTnT; dilution 1:100, Thermo Fisher Scientific, Inc.), CD31 (dilution 1:100; Abcam Plc., Cambridge, UK, or NCL-CD31-1A10, Leica Biosystems, Wetzlar, Germany), N-cadherin (dilution 1:200, Thermo Fisher Scientific, Inc.), vimentin (dilution 1:200, Abcam Plc.), and CD90 (dilution 1:200, Abcam Plc.). The secondary antibodies were 488 goat anti-rabbit IgG (H + L) (dilution 1:200) and 546 goat anti-mouse IgG (H + L) (dilution 1:200). Hoechst (dilution 1:1000) was used for nuclear staining. All images were acquired and analyzed using microscopes (BZ-X710, Keyence, Osaka, Japan).

## Results

### Analysis of hiPSC-COs and T-EHTs

**Figure 1:**
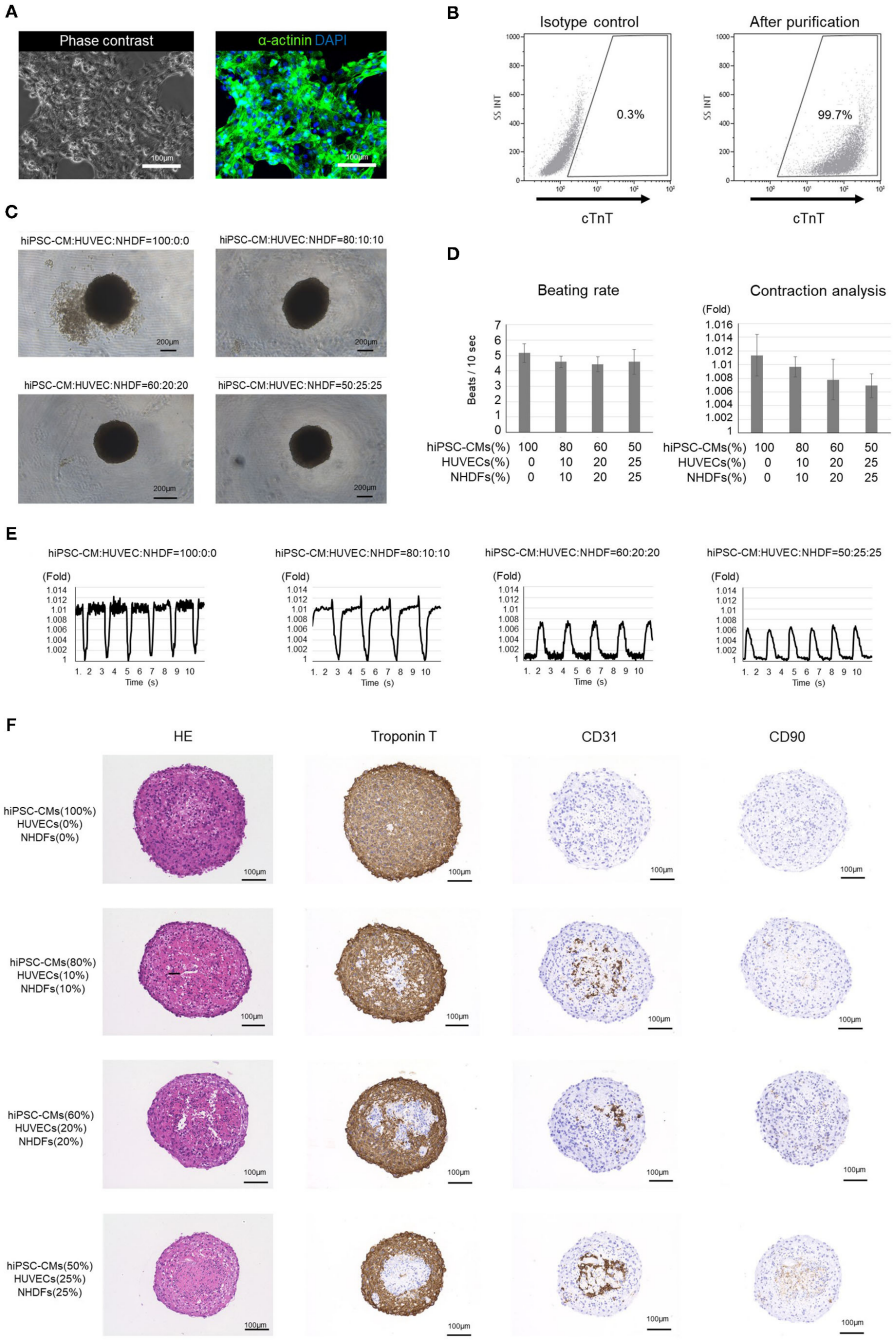
Analysis of human induced pluripotent stem cell-derived cardiomyocytes (hiPSC-CMs) and cardiac organoids (COs). **(A)** Phase contrast image and immunostaining of purified hiPSC-CMs. **(B)** Flow cytometry of purified hiPSC-CMs. The proportion of cardiac troponin T-positive cells is >99%. **(C)** Outline structure of human induced pluripotent stem cell-derived cardiac organoids (hiPSC-COs). They are composed of hiPSC-CMs, human umbilical vein endothelial cells (HUVECs), and normal human dermal fibroblasts (NHDFs) seeded in four different ratios. The hiPSC-COs comprising 50% hiPSC-CMs, 25% HUVECs, and 25% NHDFs show a perfectly circular outline. **(D)** Beating rate and contraction analysis based on a fold change compared to the minimum area. The beating rate does not vary significantly among the four groups. The extent of contraction is maximum for the hiPSC-COs composed of 100% hiPSC-CMs. **(E)** Contraction of hiPSC-COs based on a fold change compared to the minimum area. The contraction is stronger in hiPSC-COs that are composed of a higher percentage of hiPSC-CMs. **(F)** Histological staining with hematoxylin and eosin and immunohistochemical staining for cardiac troponin T, CD31, and CD90.

The hiPSC-COs developed in this experiment were formed from purified hiPSC-CMs, HUVECs, and NHDFs seeded in various ratios. Incidentally, over 99% of the purified hiPSC-CMs were cTnT-positive cardiomyocytes (Figures 1A,B). After a 10-d culture period, we observed that the COs made from the 60:20:20 and 50:25:25 mixture of hiPSC-CMs/HUVECs/NHDFs exhibited better roundness than the ones developed from the 100:0:0 and 80:10:10 mixture of hiPSC-CMs/HUVECs/NHDFs (Figure 1C). Contraction analysis revealed that the hiPSC-COs consisting of 100% hiPSC-CMs exhibited the maximum contraction, and the extent of contraction gradually decreased with the decrease in the hiPSC-CM percentage in the cell mixture (Figures 1D,E). However, the beating rate did not differ among the four groups of COs (Figure 1D).

**Figure 2:**
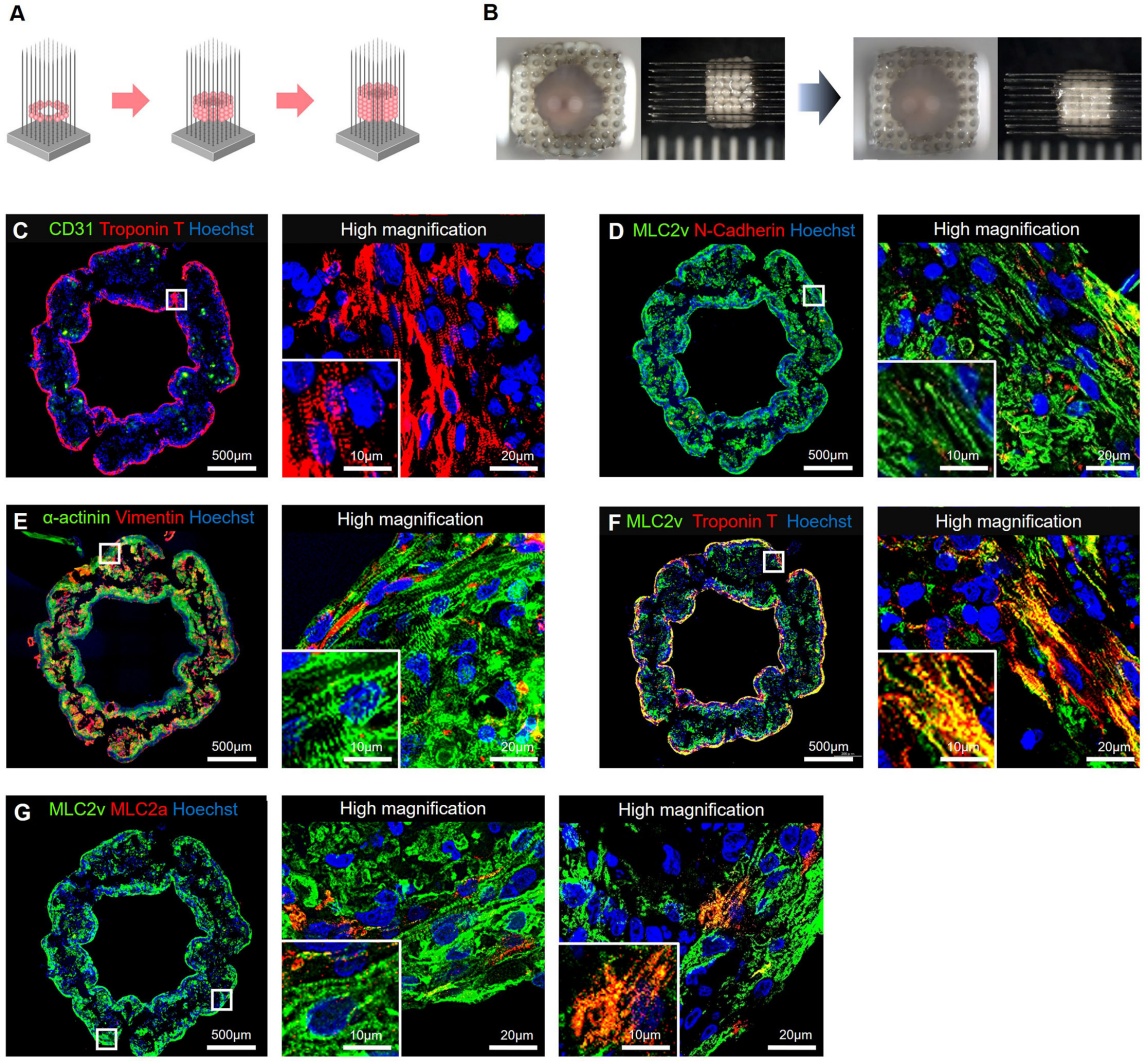
Bio-3D-printed tubular engineered heart tissues (T-EHTs). **(A)** Scheme for bio-3D printing. HiPSC-COs are placed into needles one at a time to form tubes, according to a plan. **(B)** The hiPSC-COs are cultured for 7 d, and they fused after the culture period. **(C–G)** Histological immunostaining of T-EHT and its high magnification image. **(C)** CD31 and cardiac troponin T (cTnT). **(D)** Myosin light chain 2v (MLC2v) and N-cadherin. **(E)** α-actinin and vimentin. **(F)** MLC2v and cTnT. **(G)** MLC2v and myosin light chain 2a (MLC2a). The cTnT-positive cells are located along the inner and outer boundaries of the tissue, and CD31-positive cells are in the middle **(C)**. In MLC2v, α-actinin, and cTnT staining, striations of the cardiac muscle can be clearly observed **(C–F)**. The MLC2a and MLC2v staining show a large number of MLC2v-positive cells as compared to the number of MLC2a-positive cells, and there are some MLC2a- and MLC2v-double-positive cells **(G)**.

Immunohistochemical staining showed that the cTnT-positive cells were located along the periphery, and the CD31- or CD90-positive cells were located in the middle of the hiPSC-COs (Figure 1F). Among the four types of COs, the one composed of 50% hiPSC-CMs, 25% HUVECs, and 25% NHDFs showed the highest sphericity. Both HUVECs and NHDFs have an important role in fabricating T-EHTs and engrafting them *in vivo*. We therefore used these COs to fabricate the T-EHTs. Incidentally, after the incubation period, the hiPSC-CO spheroids fused to form the tubular shape characteristic of T-EHTs (Figures 2A,B). The T-EHTs were also analyzed by histological testing. Interestingly, the cTnT-positive cells were located along the inner and outer boundaries of the tissue, while the CD31-positive cells gathered in the middle (Figure 2C), which is consistent with the observation of the hiPSC-COs (Figure 1F). As shown in Figures 2D–F, the striations of the cardiac muscles are clearly visible. Additionally, we observed vimentin-positive cells toward the middle as well as the edge of the tissue (Figure 2E). Regarding MLC2a and MLC2v staining, the tissues possessed a larger number of MLC2v-positive cells as compared to the number of MLC2a-positive cells, and some cells were double-positive for MLC2a and MLC2v (Figure 2G).

### Evaluation of T-EHT *in vivo*

**Figure 3:**
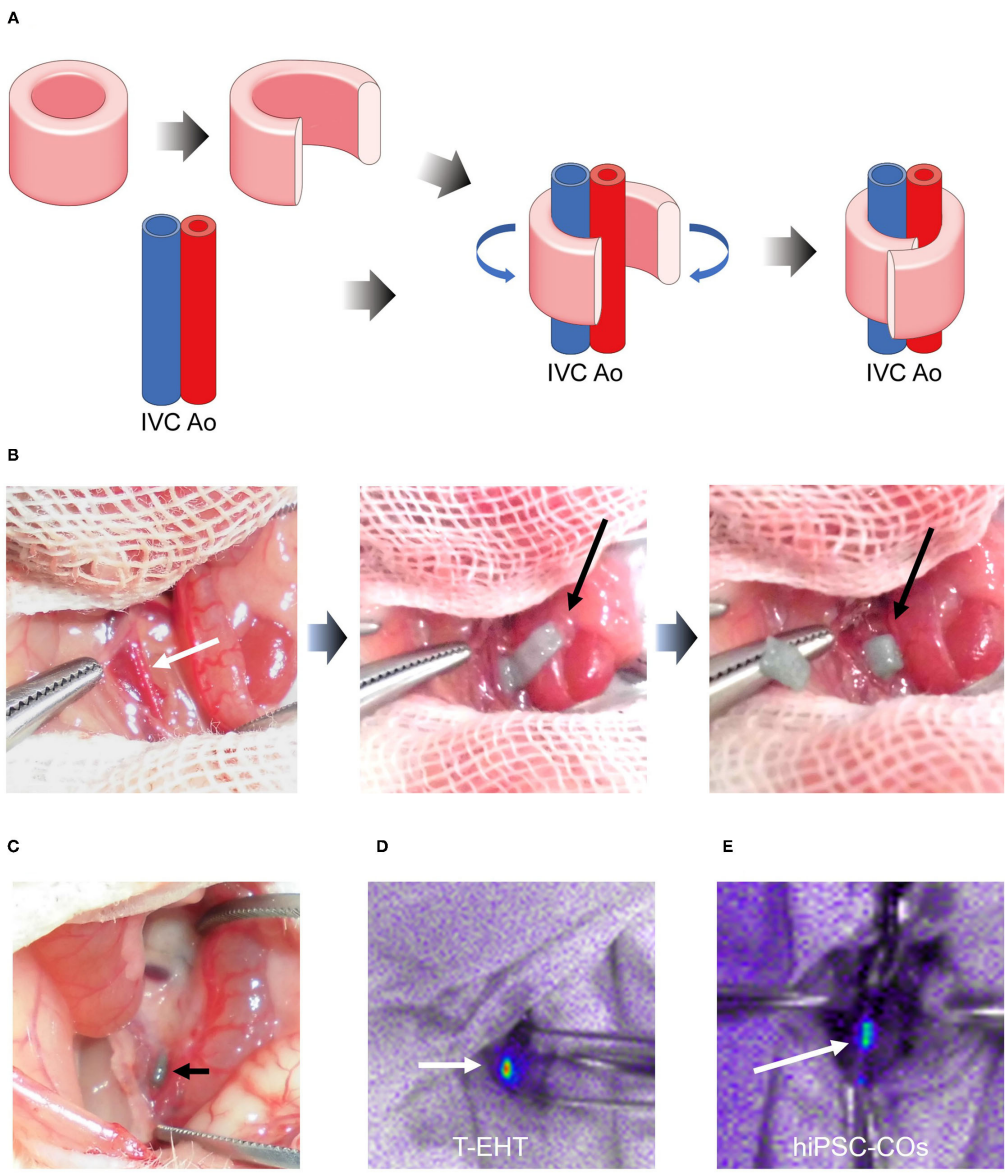
Transplantation procedure and histological analysis of transplanted tubular engineered heart tissue (T-EHT). **(A)** Scheme of transplantation procedure. The T-EHT is removed from the needle array, cut open, and wrapped around the abdominal aorta and inferior vena cava (IVC). **(B)** Transplantation procedure. The abdomen of each NOG mouse is opened under general anesthesia. The aorta and IVC are exposed (white arrow) and the T-EHT (black arrow) is wrapped around them. **(C)** After 1 w of transplantation. T-EHT (black arrow) is engrafted in the NOG mice. **(D)** and **(E)** Fluorescent images captured after 1 w of transplantation. The T-EHT and hiPSC-COs were labeled with far-red before transplantation (white arrows). Engrafted tissues with far-red fluorescence were visualized using Nightowl.

Prior to transplantation, the T-EHTs displayed spontaneous beating when they were removed from the needle array (Supplementary Video). These T-EHTs were successfully transplanted around the AA and IVC of the NOG mice and covered with the omentum (Figures 3A,B). Additionally, the hiPSC-COs were transplanted into the retroperitoneal tissues of the NOG mice and covered with the omentum (Supplementary Figure 1). The abdomen of the mice were opened 1 w post-transplantation. The T-EHT was found at the site where it had been transplanted (Figure 3C), and bioluminescent imaging displayed far-red-labeled tissue, revealing engraftment of the T-EHT and the hiPSC-COs (Figures 3D,E).

**Figure 4:**
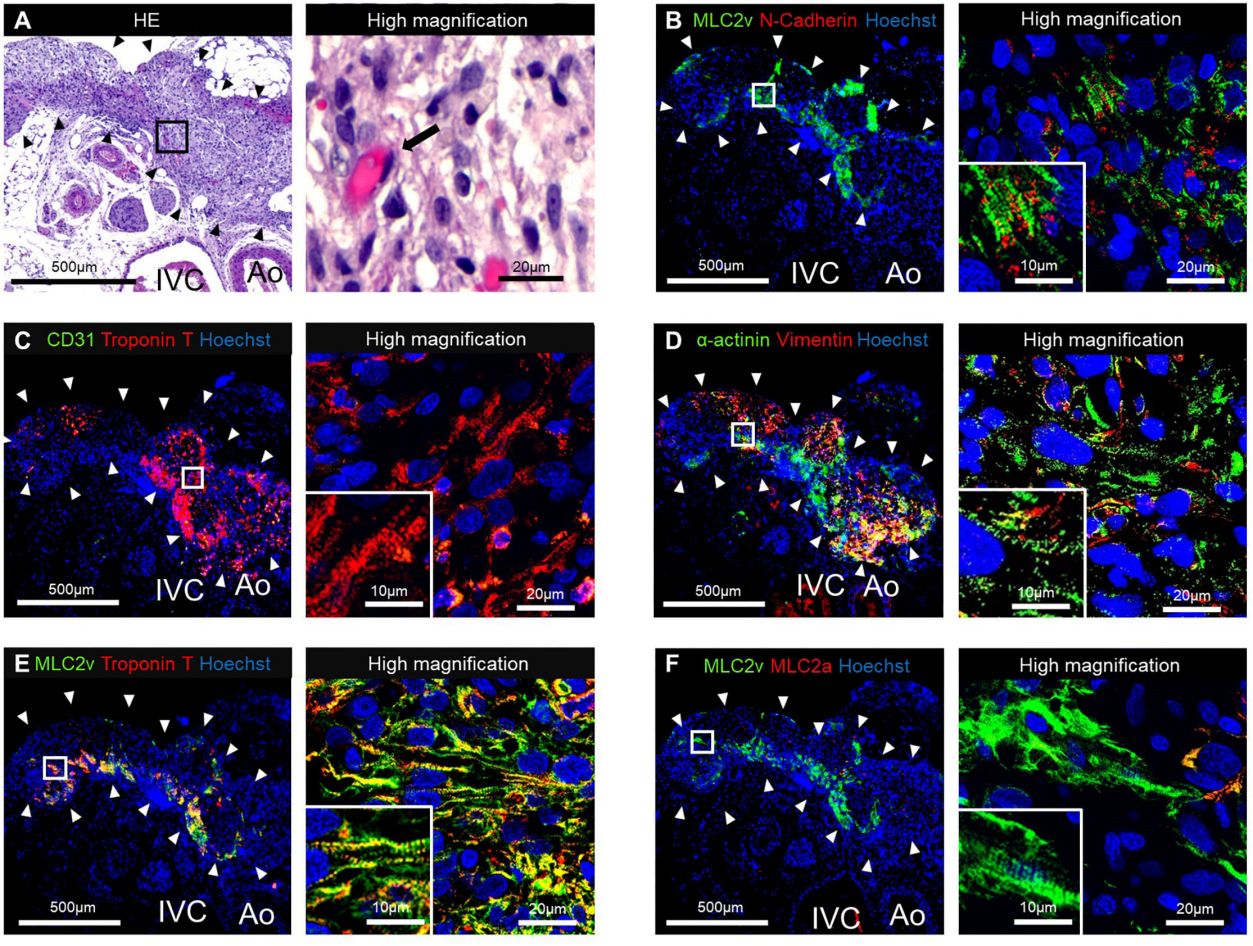
Histological staining of the tubular engineered heart tissues (T-EHTs) and its high magnifications images after 1 w of transplantation. **(A)** Hematoxylin and eosin (HE) staining. **(B)** Myosin light chain 2v (MLC2v) and N-cadherin. **(C)** CD31 and cardiac troponin T (cTnT). **(D)** α-actinin and vimentin. **(E)** MLC2v and cTnT. **(F)** MLC2v and myosin light chain 2a (MLC2a). The HE staining shows engraftment of the T-EHTs (black arrowheads) **(A)**. Red blood cells were found in the luminal structure with endothelial cells (black arrow). Immune staining reveals MLC2v-positive cells and N-cadherin-positive cells between them **(B)**. The cTnT-positive cells are found in the peripheral area of the tissue, and vimentin-positive cells are found in the middle **(C,D)**. Muscle striations are observed in MLC2a, MLC2v, and α-actinin staining **(D–F)**.

The HE staining of the resected tissue specimens showed engraftment of the T-EHT (Figure 4A). Furthermore, immunostaining revealed the presence of MLC2v- and cTnT-positive cells in the peripheral area and vimentin-positive cells in the middle of the engrafted T-EHT (Figures 4B–D). Striation of the muscles was observed in MLC2a, MLC2v, and α-actinin staining of the tissues. These striations appeared to be more distinct as compared to those observed in the subcutaneous spheroids 1 w post-transplantation (Figures 4D–F; Supplementary Figure 2).

### Engraftment of T-EHT, Functional Testing, and Histological Analysis

**Figure 5:**
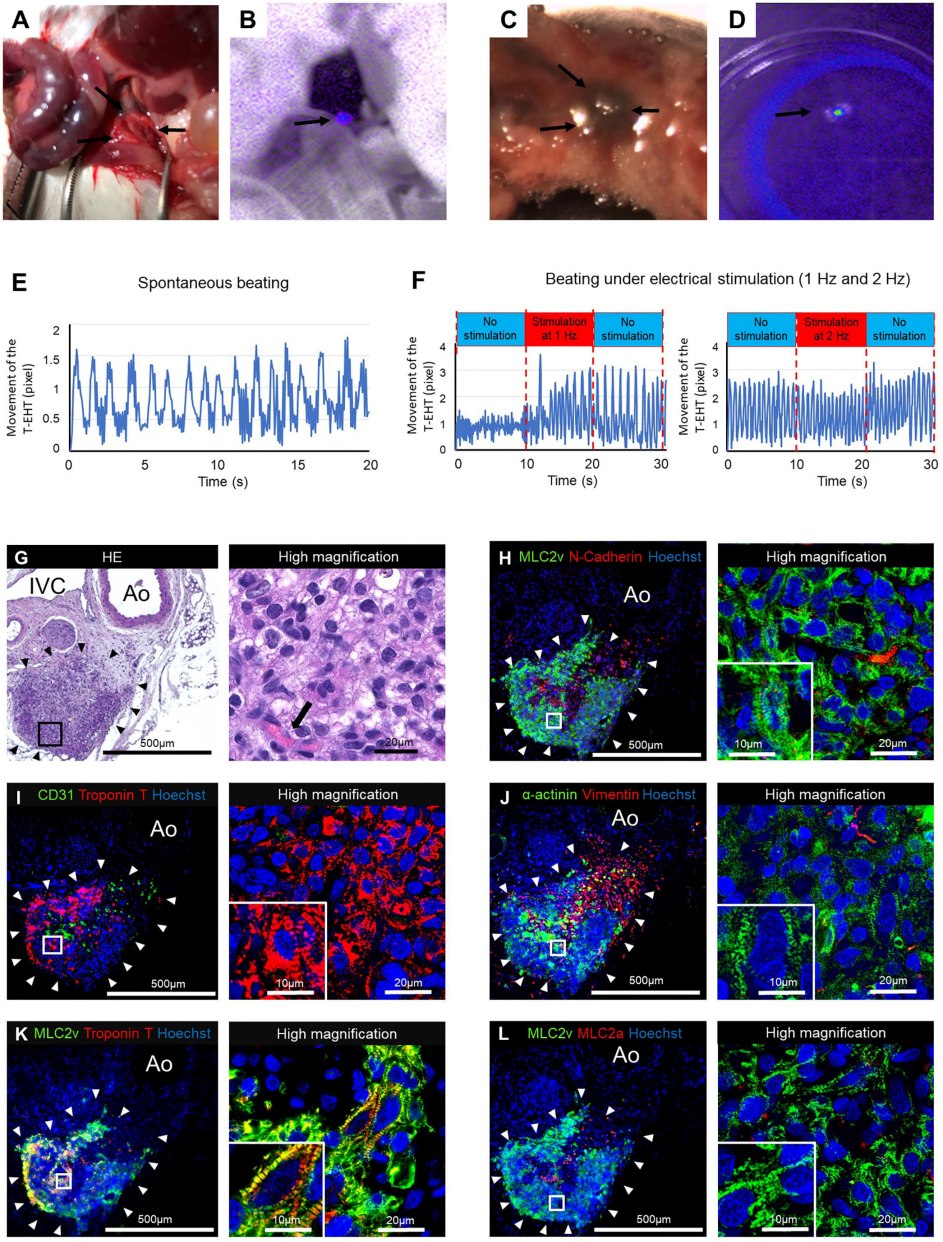
The tubular engineered heart tissues (T-EHTs) after 4 w of transplantation. **(A,B)** The T-EHT *in vivo*, highlighted in fluorescent imaging (black arrows). **(C,D)** The T-EHT is being explanted with surrounding tissues, and its fluorescence imaging record (black arrows). **(E)** Beating analysis of explanted T-EHT. The T-EHT showed spontaneous beating. **(F)** Beating analysis of explanted T-EHT with electrical stimulation. The T-EHT was stimulated with bipolar electrical pulses at 15 V and 1 Hz or 2 Hz for 10 ms. Under electrical stimulation, the beating rate increased to 1.4 Hz at 1 Hz stimulation and 1.8 Hz at 2 Hz stimulation and reduced to 1.1 Hz and 1.6 Hz after stimulation, respectively. Y-axis represents the amounts of movement (in pixels) of the T-EHT recorded using a digital camera. **(G–L)** Histological staining of T-EHT and its high magnification image. **(G)** Hematoxylin and eosin (HE) staining. **(H)** Myosin light chain 2v (MLC2v) and N-cadherin. **(I)** CD31 and cardiac troponin T (cTnT). **(J)** α-actinin and vimentin. **(K)** MLC2v and cTnT. **(L)** MLC2v and myosin light chain 2a (MLC2a). The HE staining shows engraftment of T-EHT (black arrowheads) and vascularization (black arrow) **(G)**. White arrowheads in the immune staining show T-EHT. Striation of cardiomyocytes are observed in MLC2v, cTnT, and α-actinin staining **(H–K)**. Compared to the T-EHT observed 1 w after transplantation, the number of MLC2a-positive cells is reduced 4 w after transplantation **(L)**.

The transplanted T-EHTs survived for 1 m in NOG mice after transplantation (Figure 5A). Fluorescence imaging revealed far-red-labeled tissue around the AA of the NOG mice (Figure 5B). When the T-EHTs were explanted from the mice (Figures 5C,D) and their extent of contraction was tested, we observed a spontaneous beating of the T-EHTs after incubation in a medium at 37°C (Figure 5E). Moreover, when the incubated T-EHT was transferred to the chamber connected to an electrical stimulation device (8), and bipolar electrical pulses were applied, the beating rate of the T-EHT showed an increase (Figure 5F).

The HE staining of the resected tissue specimens showed engraftment of the T-EHT around the AA and IVC of the NOG mice (Figure 5G). Additionally, immunostaining of the T-EHTs revealed the presence of MLC2v-positive cells and an N-cadherin-positive area between them (Figure 5H). The cTnT-positive cells are located in the peripheral area of the tissue (Figure 5I). Striations of the muscles can be clearly observed in the MLC2a, MLC2v, and α-actinin staining, and they appear more distinct as compared to that of the COs transplanted in the subcutaneous tissues of the mice (Figures 5J–L; Supplementary Figure 3).

## Discussion

### Experiments in NOG Mice

In this study, we first produced purified hiPSC-CMs that were grown in a 2-dimensional culture system with metabolic selection (5–7), followed by the development of spherical hiPSC-COs, and finally the production of T-EHTs. We cultured four types of hiPSC-COs, each originating from a cell mixture of hiPSC-CMs, HUVECs, and NHDFs seeded in different cell ratios. We observed that the hiPSC-COs consisting of only hiPSC-CMs (100%) exhibited strong contractions, but they were less rounded in outline than the other hiPSC-COs. In fact, this is consistent with previous studies that have reported that hiPSC-COs become rounder in outline as the proportion of hiPSC-CMs decrease (8, 12).

Both NHDFs and HUVECs are important for the development of hiPSC-COs because NHDFs promote spheroid formation and HUVECs promote vascularization (13). For fabrication of the T-EHTs, we chose hiPSC-COs consisting of 50% hiPSC-CMs, 25% HUVECs, and 25% NHDFs because they portrayed a perfectly circular outline and optimum contraction. A perfect circle is one of the essential factors in creating precise EHTs, especially while using a bio-3D printer. If the form or size of the hiPSC-COs lacks uniformity, the EHTs will have defects or pinholes in the tubular structure.

Incidentally, EHTs are of many types, such as rod-shaped, sheet-like, and tubular. The rod-shaped EHTs reportedly exhibit maturation and polarity of cells. Previously, scientists have created tubular EHTs by rolling sheet-type EHTs over a tube, resulting in engraftment and pulsation of the tube (14, 15). In the present study, we fabricated T-EHTs using a bio-3D printer with a needle array. Our T-EHTs exhibited spontaneous beating and an optimum wall thickness. In future, we can expand this method to develop a model on a larger scale than what is possible with the current T-EHTs. Moreover, we successfully fabricated the T-EHTs without using any biomaterials as the scaffold. This is a notable development because there is a strong possibility that the biomaterials will undergo degradation or generate an immune response, and their metabolites can be toxic to the host. Additionally, the hiPSC-CMs can establish better connections among themselves within a scaffold-free environment (12, 13).

Stimulation of an EHT is an important factor in its maturation process. In fact, previous studies have demonstrated the impact of electrical or physiological stimulation on the maturation of EHTs (16, 17). We believe that application of repeated stimuli to EHTs is equally important in *in vitro* and *in vivo* conditions. Therefore, we transplanted our T-EHTs around the AA of the NOG mice, so that the transplanted tissues could receive mechanical pulsation stress from the aorta. This is consistent with a previous study, which reported that wrapping cardiac cell sheets around large vessels leads to their significant maturation (18).

The T-EHTs developed in this study exhibited spontaneous beating, and they beat along with electrical pacing. In fact, our transplanted tissues could beat upon electrical stimulation even after 4 w of implantation. Since electrical stimulation helps in the maturation process, the transplanted tissue might undergo better maturation than what we observed in the present study if we could stimulate the tissue *in vivo* using a device, such as a pacemaker. Therefore, future research may explore the use of pacemakers that are synchronized with the aortic pulsation to subject the grafts to increased mechanical stress, thereby ensuring better maturation of the T-EHTs.

Vascularization is another important factor for the survival of EHTs *in vivo*. The omentum is a richly vascular organ that secretes angiogenic factors, and it is used to clinically treat patients with organ failure or infection (19). Furthermore, previous studies have reported better *in vivo* survival of hiPSC-CM cell sheets transplanted within the omentum flap (20, 21). To achieve better engraftment of the tissues in this transplantation, we covered the transplanted T-EHTs or hiPSC-COs with omentum. After 4 w of transplantation, we observed that the T-EHTs were surrounded by the omentum; hence, we believe that the omentum contributed to the engraftment of the transplanted tissue and delivered oxygen and nutrition to them. Compared to hiPSC-COs, the bio 3D-printed T-EHTs were larger in size and exhibited more prominent striations in their muscles. Previous studies have reported that the shape of the EHT affects its maturation. We observed that the tubular shape of our EHTs contributed to their maturation, similar to that observed in the rod-shaped EHTs.

### Limitations

This study has several limitations. First, we fabricated small T-EHTs that fitted around the AA of the mice. The engrafted small T-EHTs were too small and immature to assist blood circulation. Second, we did not evaluate the accurate mechanical force of the T-EHTs. Additional contraction force analysis would help us to understand the maturation of the T-EHTs (22). In the future, we intend to analyze the contraction force. Finally, we succeeded in engrafting the T-EHTs only in the NOG mice; similar transplantations were performed in nude rats, but the T-EHTs did not survive for 1 m (data not shown). This may have occurred due to immunological rejection. Therefore, future studies need to focus on overcoming these problems and on successfully transplanting a large T-EHT into a large animal model. However, creating larger and thicker T-EHTs might be challenging because cell death in the inner area of a thicker T-EHT will occur due to malnutrition. Changing the design of the T-EHT or the cultivate system could overcome this problem. We think that the ability to change the design of T-EHT is one of the advantages of using a bio-3D printer.

## Conclusion

We successfully transplanted bio-3D printed T-EHTs derived from hiPSCs into NOG mice. They exhibited spontaneous beating and were engrafted within 1 m of transplantation. However, further research is required to overcome the limitations of the present study and successfully transplant large T-EHTs in large animal models.

## Data Availability Statement

The original contributions presented in the study are included in the article/Supplementary Material, further inquiries can be directed to the corresponding author/s.

## Ethics Statement

The experimental protocol was approved by the Institutional Animal Care and Use Committee, Keio University. All experimental studies were performed in accordance with the Institutional Guidelines on Animal Experimentation at Keio University and the Fundamental Guidelines for Proper Conduct of Animal Experiments and Related Activities in Academic Research Institutions (Ministry of Education, Culture, Sports, Science and Technology). Written informed consent was obtained from the owners for the participation of their animals in this study.

## Author Contributions

YK, ST, KA, KN, and EK designed the study. YK performed and analyzed most of the experiments. YK, ST, KA, YS, and EK performed all animal procedures. YK and KA performed the histological analysis. ST and KF produced purified cardiomyocytes from hiPSCs. KA, TT, and KN fabricated the cardiac organoids and tubuar engineered heart tissues. YK, ST, KA, YS, and HS analyzed all other data and provided administrative assistance. YK and ST wrote the original draft. ST, KN, and EK reviewed and edited the manuscript. ST acquired funding. ST, HS, KF, and KN supervised the study. All authors contributed to the article and approved the submitted version.

## Funding

This work was supported by the Japan Society for the Promotion of Science (JSPS) KAKENHI 20H03768 to ST.

## Conflict of Interest

KF is a co-founder and CEO of Heartseed Inc. ST and HS is an advisor of Heartseed, Inc. ST, HS, and KF own equity in Heartseed, Inc. KN is a co-founder and shareholder of Cyfuse Biomedical KK and an inventor/developer designated on the patent for the Bio-3D printer. The remaining authors declare that the research was conducted in the absence of any commercial or financial relationships that could be construed as a potential conflict of interest.

## Publisher's Note

All claims expressed in this article are solely those of the authors and do not necessarily represent those of their affiliated organizations, or those of the publisher, the editors and the reviewers. Any product that may be evaluated in this article, or claim that may be made by its manufacturer, is not guaranteed or endorsed by the publisher.

## Supplementary Material

Supplementary Video The tubular engineered heart tissue (T-EHT) was removed from the needle array and cut open. The T-EHT, which was cut open, showed spontaneous beating.

Supplementary Figure 1 Transplantation of human induced pluripotent stem cell-derived cardiac organoids (hiPSC-COs) into mouse abdomen. The abdomen of the mouse was opened under general anesthesia. The abdominal aorta (AA) and the inferior vena cava (IVC) were exposed. The hiPSC-COs were seeded around the AA and the IVC and covered with omentum. Black arrowheads shows hiPSC-COs.

Supplementary Figure 2 Histological staining of the hiPSC-COs in subcutaneous tissue and its high magnification image after 1 w of transplantation. **(A)** Hematoxylin and eosin (HE) staining. **(B)** Myosin light chain 2v (MLC2v) and N-cadherin. **(C)** CD31 and cardiac troponin T (cTnT). **(D)** α-actinin and vimentin. **(E)** MLC2v and cTnT. **(F)** MLC2v and MLC2a. The HE staining shows engraftment of the hiPSC-COs. Immunohistochemical staining reveals cTnT-positive cells, MLC2v-positive cells, MLC2a-positive cells, and α-actinin-positive cells, but striations are less prominent than those of T-EHTs (shown in Figure 4).

Supplementary Figure 3 Histological staining of the hiPSC-COs 1 m after transplantation into the subcutaneous tissue on the back of the NOG mice. **(A)** Hematoxylin and eosin (HE) staining. **(B)** Myosin light chain 2v (MLC2v) and N-cadherin. **(C)** CD31 and cardiac troponin T (cTnT). **(D)** α-actinin and vimentin. **(E)** MLC2v and cTnT. **(F)** MLC2v and MLC2a.Similar to the T-EHTs, HE staining shows engraftment of the hiPSC-COs (black arrowhead), and there is vascularization (A). White arrowheads show hiPSC-COs. The striations of the cardiomyocytes are observed in MLC2v and cTnT staining, but compared to the striations of the T-EHTs, the striations appear unclear **(B–F)**.

## Abbreviations

AA: abdominal aorta
cTnT: cardiac troponin T
EHT: engineered heart tissue
hiPSC: human induced pluripotent stem cell
hiPSC-CM: human induced pluripotent stem cell-derived cardiomyocyte
hiPSC-CO: human induced pluripotent stem cell-derived cardiac organoid
HUVEC: human umbilical vein endothelial cell
IVC: inferior vena cava
MLC2a: myosin light chain 2a
MLC2v: myosin light chain 2v
NHDF: normal human dermal fibroblast
T-EHT: tubular engineered heart tissue.
