# Supplementary figures and images for: Scaffold-Free Tubular Engineered Heart Tissue From Human Induced Pluripotent Stem Cells Using Bio-3D Printing Technology in vivo
Source: Front Cardiovasc Med. 2022 Jan 20;8:806215. doi: 10.3389/fcvm.2021.806215 (PMC8811174; doi:10.3389/fcvm.2021.806215)

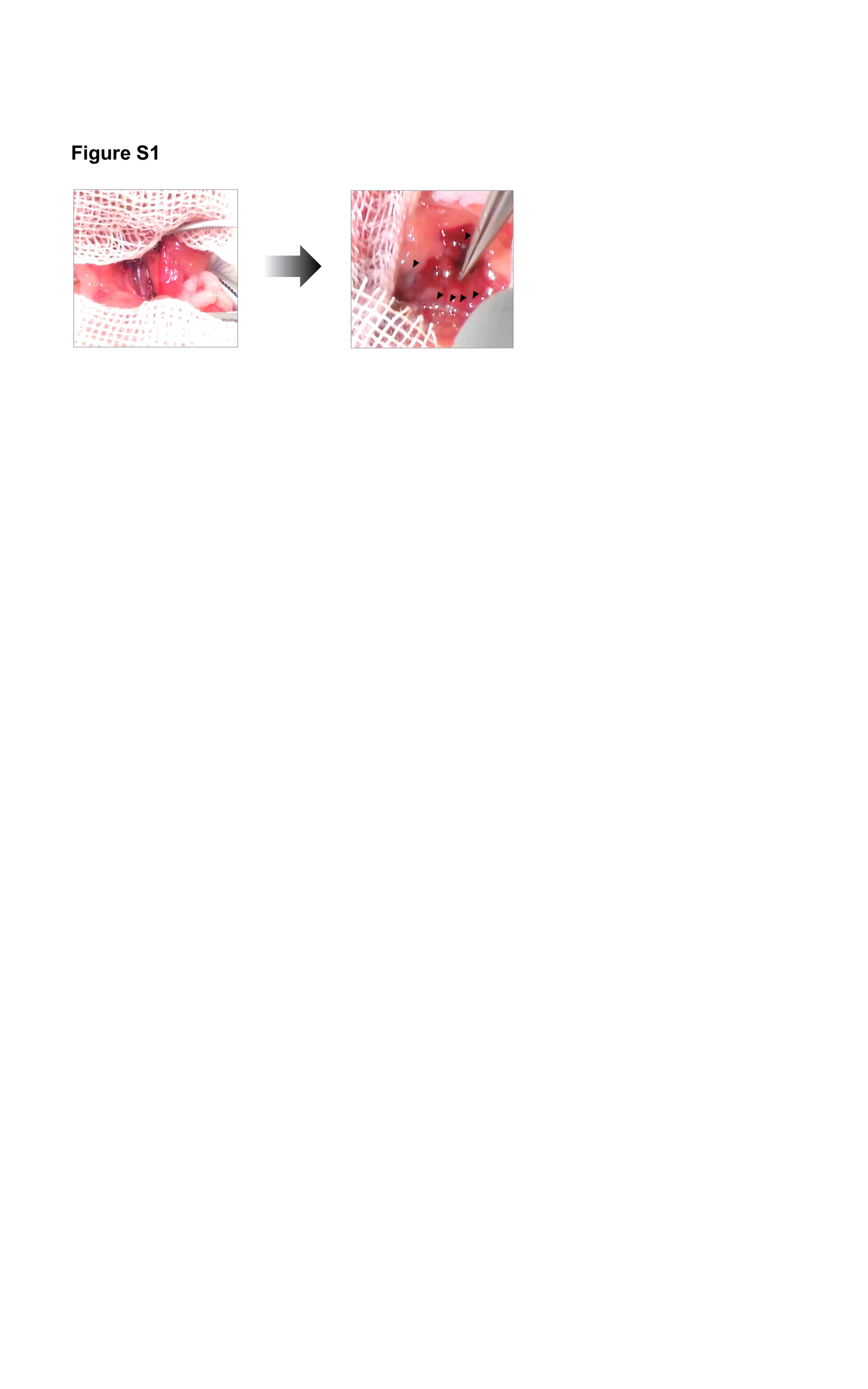

Supplement: Supplementary Figure 1 — Transplantation of human induced pluripotent stem cell-derived cardiac organoids (hiPSC-COs) into mouse abdomen. The abdomen of the mouse was opened under general anesthesia. The abdominal aorta (AA) and the inferior vena cava (IVC) were exposed. The hiPSC-COs were seeded around the AA and the IVC and covered with omentum. Black arrowheads shows hiPSC-COs. [file Image_1.JPG]

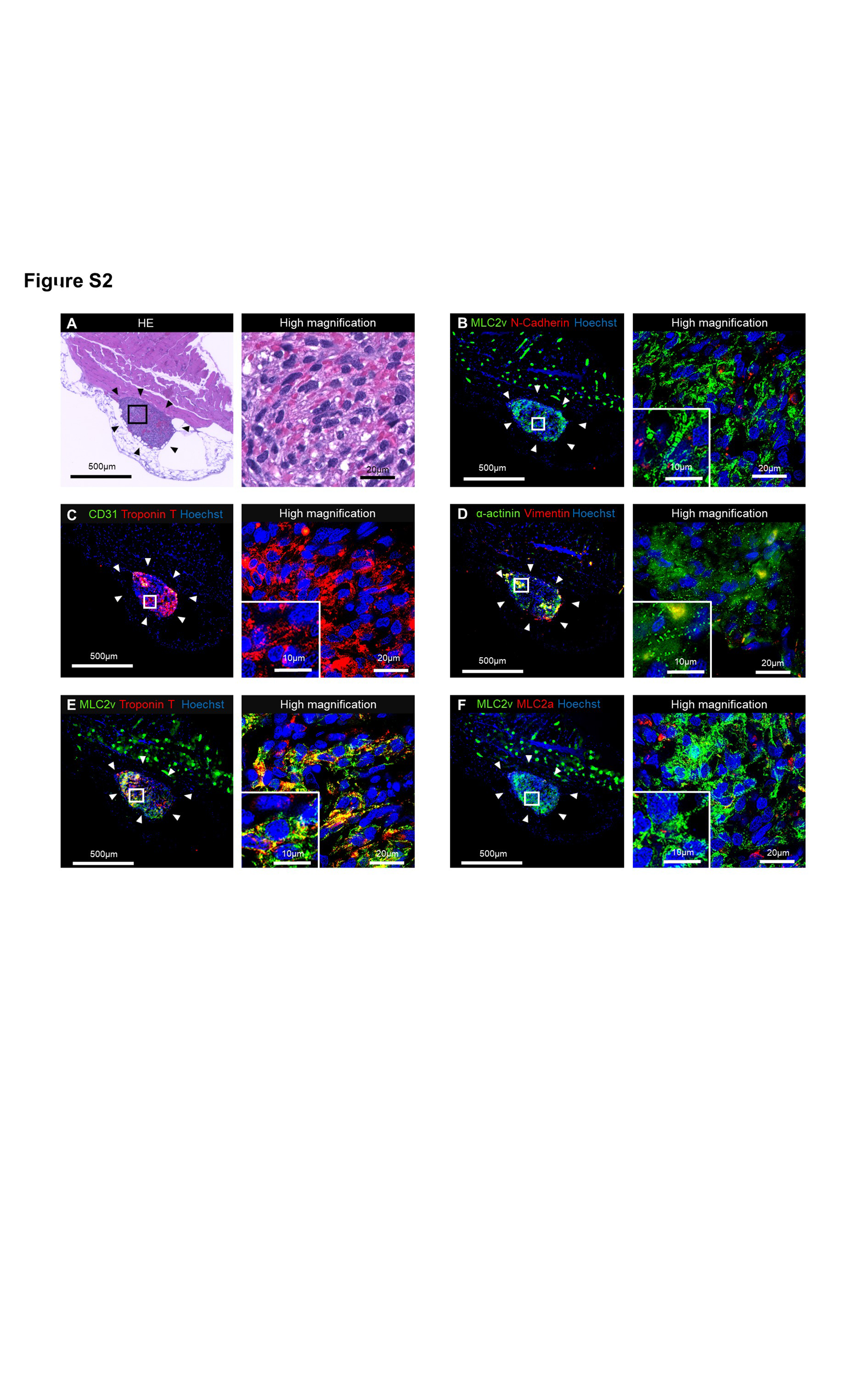

Supplement: Supplementary Figure 2 — Histological staining of the hiPSC-COs in subcutaneous tissue and its high magnification image after 1 w of transplantation. (A) Hematoxylin and eosin (HE) staining. (B) Myosin light chain 2v (MLC2v) and N-cadherin. (C) CD31 and cardiac troponin T (cTnT). (D) α-actinin and vimentin. (E) MLC2v and cTnT. (F) MLC2v and MLC2a. The HE staining shows engraftment of the hiPSC-COs. Immunohistochemical staining reveals cTnT-positive cells, MLC2v-positive cells, MLC2a-positive cells, and α-actinin-positive cells, but striations are less prominent than those of T-EHTs (shown in Figure 4). [file Image_2.JPG]

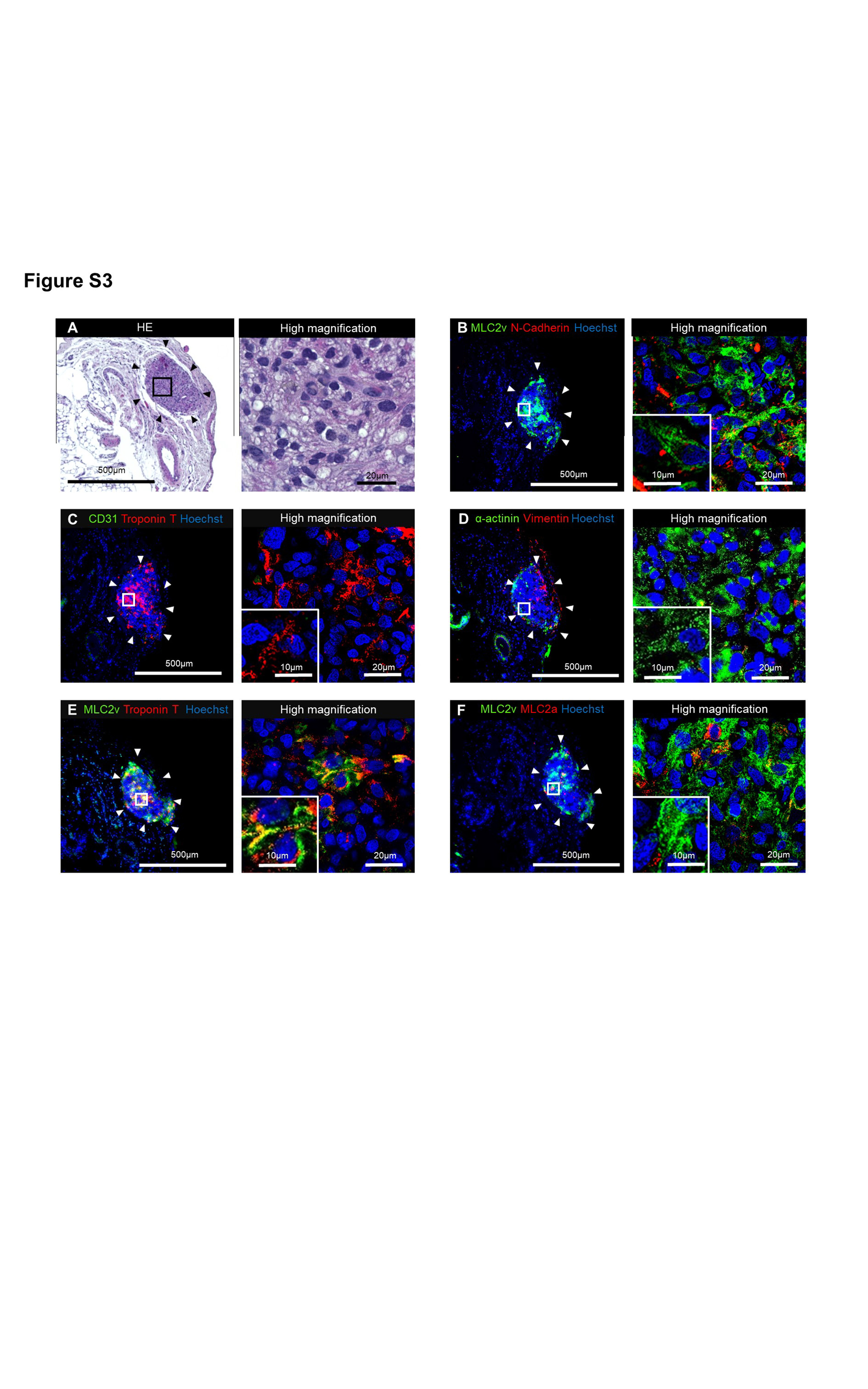

Supplement: Supplementary Figure 3 — Histological staining of the hiPSC-COs 1 m after transplantation into the subcutaneous tissue on the back of the NOG mice. (A) Hematoxylin and eosin (HE) staining. (B) Myosin light chain 2v (MLC2v) and N-cadherin. (C) CD31 and cardiac troponin T (cTnT). (D) α-actinin and vimentin. (E) MLC2v and cTnT. (F) MLC2v and MLC2a.Similar to the T-EHTs, HE staining shows engraftment of the hiPSC-COs (black arrowhead), and there is vascularization (A). White arrowheads show hiPSC-COs. The striations of the cardiomyocytes are observed in MLC2v and cTnT staining, but compared to the striations of the T-EHTs, the striations appear unclear (B–F). [file Image_3.JPG]
